# Large Asymmetric Hypertrophy of Rectus Abdominis Muscle in Professional Tennis Players

**DOI:** 10.1371/journal.pone.0015858

**Published:** 2010-12-31

**Authors:** Joaquin Sanchis-Moysi, Fernando Idoate, Cecilia Dorado, Santiago Alayón, Jose A. L. Calbet

**Affiliations:** 1 Department of Physical Education, University of Las Palmas de Gran Canaria, Las Palmas de Gran Canaria, Spain; 2 Radiology Department, Clínica San Miguel, Pamplona, Spain; 3 Diagnostic Imaging Department, Hospital San Roque Maspalomas, Grupo San Roque, Maspalomas (Gran Canaria), Spain; Universidad Europea de Madrid, Spain

## Abstract

**Purpose:**

To determine the volume and degree of asymmetry of the musculus *rectus abdominis* (RA) in professional tennis players.

**Methods:**

The volume of the RA was determined using magnetic resonance imaging (MRI) in 8 professional male tennis players and 6 non-active male control subjects.

**Results:**

Tennis players had 58% greater RA volume than controls (P = 0.01), due to hypertrophy of both the dominant (34% greater volume, P = 0.02) and non-dominant (82% greater volume, P = 0.01) sides, after accounting for age, the length of the RA muscle and body mass index (BMI) as covariates. In tennis players, there was a marked asymmetry in the development of the RA, which volume was 35% greater in the non-dominant compared to the dominant side (P<0.001). In contrast, no side-to-side difference in RA volume was observed in the controls (P = 0.75). The degree of side-to-side asymmetry increased linearly from the first lumbar disc to the pubic symphysis (r = 0.97, P<0.001).

**Conclusions:**

Professional tennis is associated with marked hypertrophy of the musculus *rectus abdominis*, which achieves a volume that is 58% greater than in non-active controls. *Rectus abdominis* hypertrophy is more marked in the non-dominant than in the dominant side, particularly in the more distal regions. Our study supports the concept that humans can differentially recruit both *rectus abdominis* but also the upper and lower regions of each muscle. It remains to be determined if this disequilibrium raises the risk of injury.

## Introduction

Tennis is an asymmetric sport causing marked muscle hypertrophy in the dominant arm compared to the non-dominant arm [1], [2]. During every tennis stroke, the arm that holds the racket is only the last link of a kinetic chain involving the sequential activation of the trunk muscles to cause trunk rotation and flexion movements to facilitate the transfer moment from the legs and trunk to the arm and the racket [3]. This implies that the recruitment of the trunk muscles is also asymmetric [4]. However, it remains unknown whether tennis elicits an asymmetric hypertrophy of the abdominal muscles.

*Rectus abdominis* (RA) is considered the main responsible of trunk flexion [5]. In tennis players, RA plays an important role for power generation in every stroke, but particularly when serving [4]. The serve is preceded by a lumbar extension followed by a powerful trunk flexion and rotation to the direction of the non-dominat side [3]. In the last part of the movement, the contralateral RA muscle registers higher electromyografic activity compared to the dominant RA [4]. *Rectus abdominis* functional capacities depend on sport practice [6]. Studies using isokinetic machines have shown strength differences in trunk flexion between competitive tennis players and non-active controls [7]–[9]. Tennis players develop greater strength during non-dominant than dominant lateral trunk flexion and show greater strength during trunk flexion than extension. In contrast, non-active controls have higher strength during trunk extension and balanced dominant/non-dominant lateral flexion strength ratios [7]–[9].

*Rectus abdominis* muscle strains and lower back pain are frequent in elite tennis players [10]. Traditionally, these injuries have been associated to side-to-side strength differences, as well as to strength unbalances between abdominal and back extensor muscles [11]–[16]. Cross-sectional magnetic resonance images (MRI) and ultrasonographic exams have shown that most RA muscle strains occur in the distal rectus, below the umbilicus [17], [18]. At this level, RA hypertrophy is greater in the non-dominant than in the dominant side [17], [18]. In contrast, ultrasound images revealed symmetric RA cross sectional areas (CSA) and thickness above the umbilicus region in moderate active male and female subjects [19].

The main aim of this study was to determine the volume and degree of asymmetry of the musculus *rectus abdominis* (RA) in professional tennis players compared to non-active controls. A secondary aim was to localize the level at which the magnitude of asymmetry, as reflected by the cross-sectional area (CSA), is greater.

The hypothesis to be tested is that professional tennis is associated with an asymmetric development of the *rectus abdominis* muscle, with greater volume in the non-dominant compared to the dominant side, reflecting greater stretch-shortening loads during tennis actions on the non-dominant *rectus abdominis*.

## Methods

### Subjects

Eight male professional tennis players and 6 non-athletes (control group: CG) agreed to participate in the study (Table 1). Participants of the CG had never been involved in regular physical exercise. All participants were informed about the potential benefits and risks of the study and gave a written consent to participate. The study was approved by the ethical committee of the University of Las Palmas de Gran Canaria. All tennis players started tennis practice before 12 years old and had been training and participating in professional tennis competitions of the International Tennis Federation (Futures and Challengers tournaments). Their current dedication to tennis was 25±7 h/week. Six tennis players were right handed and two of them used the two hands backhand stroke. The two left handed players used a one hand backhand stroke. In this article the dominant side of the RA corresponds to the same side of the dominant arm, and vice versa.

**Table 1:** Physical characteristics of tennis players and control group, and total and regional length of *rectus abdominis* from pubic symphysis to the discal space between L1 and L2 (mean ± SD).

Variables	Tennis	Controls
Age (years)	21.9±3.8	27.5±8.1
Height (cm)	182.5±3.9	177.7±2.6^a^
Body mass (Kg)	75.4±6.9	75.5±11.1
BMI (Kg/m^2^)	22.6±1.5	23.9±3.5
*Rectus abdominis* length (cm)		
1^st^ segment	3.5±0.5	3.7±0.5
2^nd^ segment	3.0±0.0	3.0±0.0
3^rd^ segment	3.8±0.4	3.2±0.5^a^
4^th^ segment	3.3±0.4	2.8±0.5
5^th^ segment	3.6±0.5	3.3±0.5
6^th^ segment	3.0±0.0	3.0±0.0
7^th^ segment	3.9±0.4	3.0±0.0^b^
8^th^ segment	3.1±0.6	2.8±0.4
Total	27.1±2.2	25.2±1.8

a P = 0.03 CG vs. TP,

b P<0.001 CG vs TP.

### Magnetic resonance imaging

Magnetic resonance imaging (MRI) was used to determine the muscle CSA and muscle volume of the left and right RA. A 1.5 T MRI scanner (Philips Achieva 1.5 Tesla system, Philips Healthcare, Best, the Netherlands) was used to acquire 10-mm axial contiguous slices from trunk, abdomen and pelvis, i.e., without interslice separation. Sagittal, coronal and transverse localizers of the body were obtained to determine precisely the anatomic sites for image acquisition. Transverse MRI images at rest (a breath-hold at mid expiration) oriented to be perpendicular to the anterior abdominal wall were obtained. Axial gradient-echo T1-weighted MR images was used with a repetition time of 132 ms and an echo time of 4.2 ms, flip-angle of 80° with a 42 cm^2^ field of view and a matrix of 256×256 pixels (in-plane spatial resolution 1.64 mm×1.64 mm). The body coil was used for image acquisition. The total research time was about 20 seconds which was within the breath-hold tolerance of all participants.

**Figure 1:**
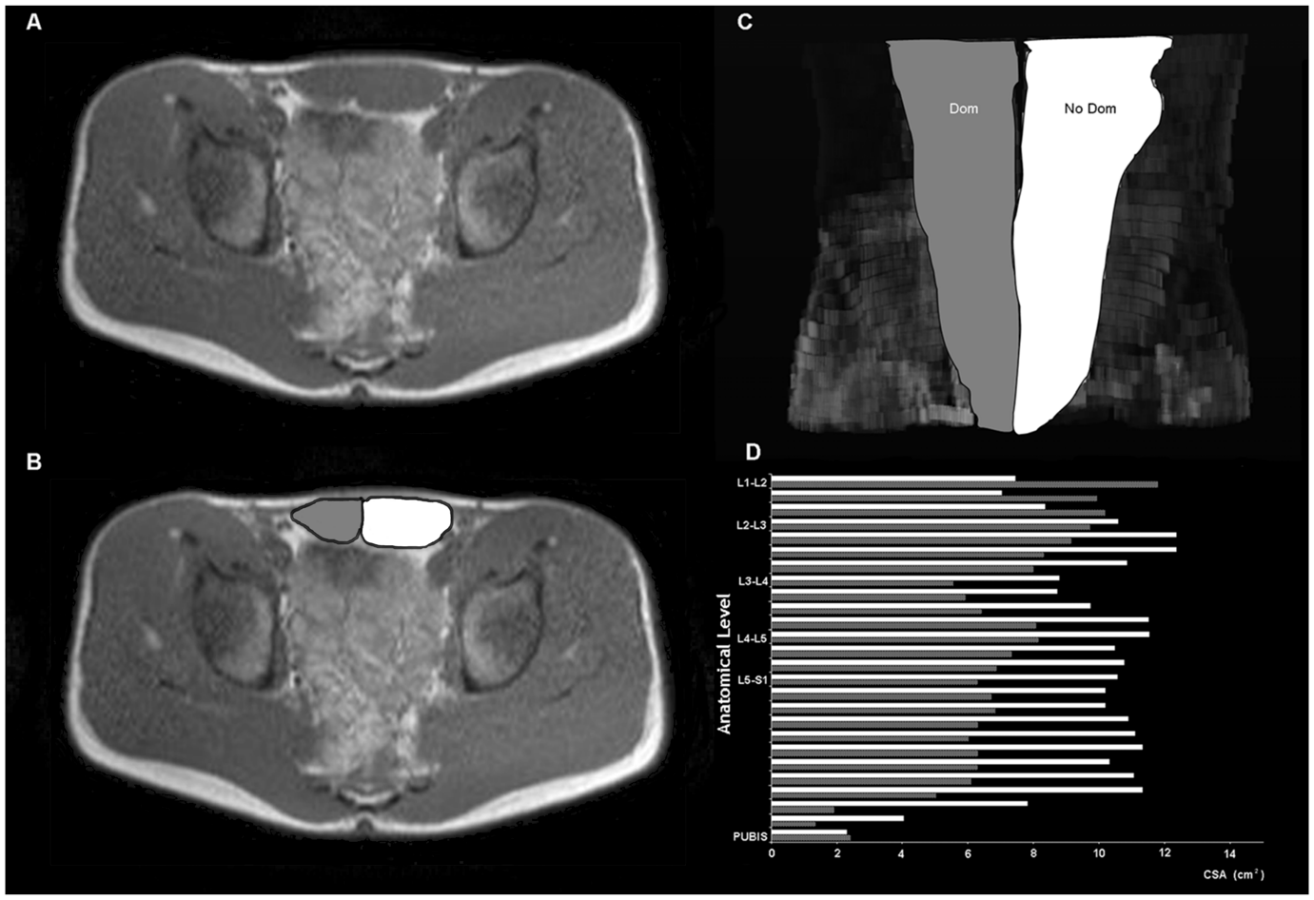
Digital reconstruction of *rectus abdominis* muscle of one right-handed professional tennis player, from magnetic resonance images (MRI). (A) Cross sectional MRI at the umbilical level, and (B) corresponding image showing the different muscle compartments measured. (C) Digital reconstruction of *rectus abdominis* muscle in the coronal plane, from L1-L2 to the pubic symphysis, and (D) figure illustrating the successive cross sectional MRI measurements performed. In gray, the dominant side (Dom), in white, the non dominant side (NoDom) of *rectus abdominis* muscle.

The acquired MRI images were transferred to a computer for digital reconstruction to determine the CSA (Fig. 1). The muscle volumes were calculated between L1-L2 discal level and the pubic symphysis. Each image was labeled referred to discal spaces, cranial aspect of coxofemoral joint and pubic symphysis using sagittal and axial scout images. All calculations were carried out by the same investigator, who was blinded to arm dominance, using a specially designed image analysis software (SliceOmatic 4.3, Tomovision Inc., Montreal, Canada), as described elsewhere [20]. A threshold was selected for adipose and lean tissues on the basis of the grey-level image pixel histograms to identify tissue area and the tissue boundaries were manually traced [20].

The total volume (V_total_) of the RA was assessed in each participant [21]. Regional RA volumes were also calculated for comparative purposes. V_total_ was divided into 8 regions (S, segments) (S_1_ to S_8_, from proximal to distal). To determine the boundaries of each segment the total number of slices was divided by 2. Then, each fraction was divided successively by 2 until 8 segments were obtained. Then the volume of each segment was calculated using the same procedures described to calculate V_total_. If the initial number of slices in any fraction was odd, the criteria used to include the extra slice after every division by 2 was to include it into the most distal region (Table 2). Tendinous inscriptions were distributed symmetrically in all subjects, i.e they lied at the same height in the right and left RA.

**Table 2:** Example of method used to divide the *rectus abdominis* muscle into segments and the slices included in each segment to calculate the corresponding volume.

Anatomical region	Muscle length (mm)	Number of slices	1^st^ division	2^nd^ division	3^rd^ division	Segment
**Pubic Symphysis**	10	Slice 1	Slice 1	Slice 1	Slice 1	S1
	20	Slice 2	Slice 2	Slice 2	Slice 2	
	30	Slice 3	Slice 3	Slice 3	Slice 3	
	40	Slice 4	Slice 4	Slice 4	Slice 4	
	50	Slice 5	Slice 5	Slice 5	Slice 1	S2
	60	Slice 6	Slice 6	Slice 6	Slice 2	
	70	Slice 7	Slice 7	Slice 7	Slice 3	
	80	Slice 8	Slice 8	Slice 1	Slice 1	S3
	90	Slice 9	Slice 9	Slice 2	Slice 2	
	100	Slice 10	Slice 10	Slice 3	Slice 3	
	110	Slice 11	Slice 11	Slice 4	Slice 4	
	120	Slice 12	Slice 12	Slice 5	Slice 1	S4
	130	Slice 13	Slice 13	Slice 6	Slice 2	
	140	Slice 14	Slice 14	Slice 7	Slice 3	
	150	Slice 15	Slice 1	Slice 1	Slice 1	S5
	160	Slice 16	Slice 2	Slice 2	Slice 2	
	170	Slice 17	Slice 3	Slice 3	Slice 3	
	180	Slice 18	Slice 4	Slice 4	Slice 4	
	190	Slice 19	Slice 5	Slice 5	Slice 1	S6
	200	Slice 20	Slice 6	Slice 6	Slice 2	
	210	Slice 21	Slice 7	Slice 7	Slice 3	
	220	Slice 22	Slice 8	Slice 1	Slice 1	S7
	230	Slice 23	Slice 9	Slice 2	Slice 2	
	240	Slice 24	Slice 10	Slice 3	Slice 3	
	250	Slice 25	Slice 11	Slice 4	Slice 1	S8
	260	Slice 26	Slice 12	Slice 5	Slice 2	
L1/L2	270	Slice 27	Slice 13	Slice 6	Slice 3	

### Statistical analysis

Results are presented as means ± standard deviation, except for the bar figures which are presented as means ± standard error of the mean. Side-to-side comparisons were carried out using the paired Student's t-test adjusted for multiple comparisons using the Bonferroni-Holm method. Analyses of covariance were performed to compare differences across groups, with age, BMI (body mass index) and total length of *rectus abdominis* muscle as covariates. Between-groups segment-to-segment comparisons were adjusted for the length of segment under scrutiny. The relationship between muscle length and muscle volumes or CSAs into each group was determined by linear regression analysis. To test the similarity of slopes and intercepts of these relationships, the corresponding t-test was applied for the model: Y_ij_ = α_i_+ β_i_X_ij_ + ε_ij_ for i = 1,2 (1 =  tennis players, 2 =  controls) and j = 1,…, n_1_ being ε_ij_ i.i.d. random variables following a distribution N(0, σ_1_). SPSS package (SPSS Inc., Chicago, IL, USA, v15.0) for personal computers was used for the statistical analysis. Significant differences were assumed when P<0.05.

## Results

### Physical characteristics and length of rectus abdominis

Physical characteristics and total and regional length of rectus abdominis muscle are summarized in Table 1. Tennis players and controls were comparable in age and body mass. Tennis players were significantly taller than controls (P = 0.03) but the length of the rectus abdominis was not significantly different (27.1±2.2 vs. 25.2±1.8 cm, for TP and CG respectively, P = 0.10).

### Differences into each group

#### Muscle volumes

Table 3 summarizes total and regional muscle volumes in tennis players and controls. In tennis players the total volume of the non-dominant side was 35% greater compared to the dominant side (P<0.001), due to muscle hypertrophy in all segments (Table 3). In contrast, no side-to-side differences in total volume were observed in the control group (P = 0.75). In controls, the non-dominant segments 2, 7 and 8 were hypertrophied compared to the dominant side (Table 3), whilst side-to-side differences were not statistically significant at the other segmental levels.

**Table 3:** Total and regional *rectus abdominis* muscle volumes (values expressed in cm^3^, mean ± SD) and asymmetries.

Segments	Tennis Players					Controls				
	Dominant	Non-dominant		Total	Asymmetry(%)	Dominant	Non-dominant		Total	Asymmetry(%)
S1	28.5±9.4	33.1±10.4	*P = 0.047*	61.6±19.1	18	20.0±3.3	20.8±5.4	*P = 0.57*	40.8±8.3	4
S2	32.1±4.3	40.6±10.1	*P = 0.02*	72.8±13.6	26	23.1±6.1	20.0±6.3	*P = 0.02*	43.0±12.2	-14
S3	21.6±6.4	28.6±5.2	*P<0.001*	50.1±11.4	36	20.6±4.3	18.8±2.7	*P = 0.49*	39.4±4.3	-4
S4	30.1±6.0	42.1±14.1	*P = 0.008*	72.1±19.6	38	23.6±4.5	22.0±6.5	*P = 0.26*	45.6±10.7	-8
S5	27.3±5.1	39.6±8.8	*P = 0.001*	66.9±13.2	46	20.5±4.3	21.6±4.8	*P = 0.29*	42.1±8.8	5
S6	32.5±7.2	46.3±13.5	*P = 0.002*	78.9±20.2	42	21.9±3.1	23.4±4.3	*P = 0.17*	45.3±7.1	7
S7	22.5±5.6	33.7±10.5	*P = 0.002*	56.2±15.4	51	19.0±2.4	20.7±2.0	*P = 0.009*	39.7±4.3	10
S8	9.2±3.9	13.6±5.6	*P = 0.008*	22.9±9.0	55	9.2±5.7	11.7±6.3	*P = 0.004*	20.9±11.9	34
Total	205.0±35.8	277.3±67.4	*P<0.001*	482.3±101.6	35	157.7±23.8	159.0±27.0	*P = 0.75*	316.7±67.7	1

Comparisons are made between dominant and non-dominant sides into each group.

**Figure 2:**
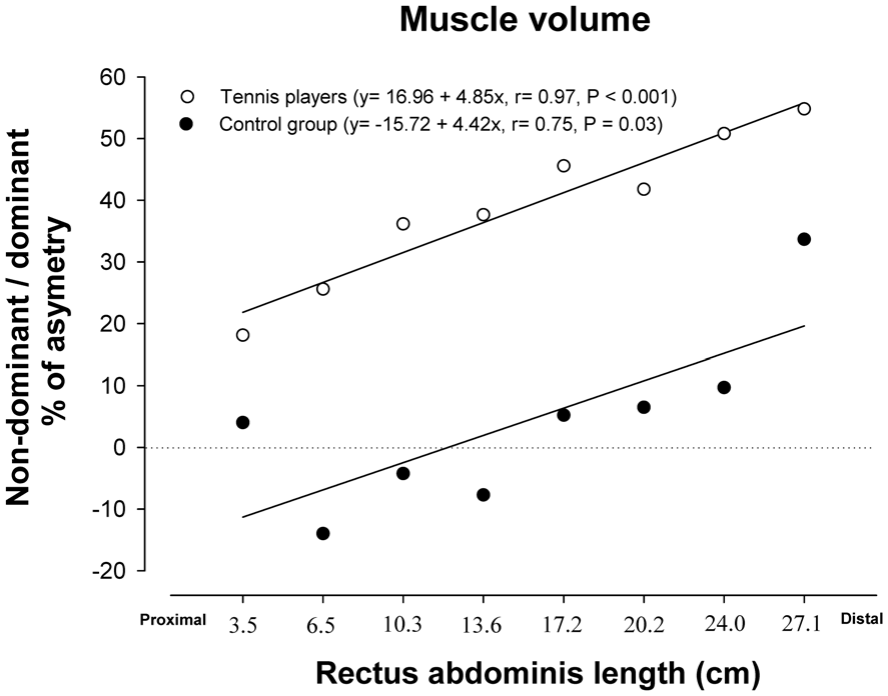
Relationship between the asymmetry in muscle volume of the dominant and non-dominant sides (expressed in percentage) and the *rectus abdominis* segments ordered in the rostro-caudal direction. In professional tennis players (white circles) and non-active subjects (black circles). Not significant differences were observed between the slopes, while the intercepts were significantly different.

A positive relationship was observed between muscle length starting from the inter-discal L1-L2 space and the degree of asymmetry in muscle volume expressed as the non-dominant/dominant ratio in TP (r = 0.97, P<0.001) and in controls (r = 0.75, P = 0.03), being more asymmetric the more distal segments (Fig. 2).

#### Cross sectional area (CSA)

Table 4 summarizes the maximum CSA into each segment. In tennis players, the non-dominant side had greater CSA than the dominant side in all segments. In controls, segments 7 and 8 had a greater CSA in the non-dominant than in the dominant side, whilst no side-to-side differences were observed in segments 1 to 6 (Table 4). In tennis players, the maximum CSA was located more distally in the non-dominant compared to the dominant side (12.6±8.5 vs. 22.9±1.2 cm from the pubic symphysis, respectively, P = 0.01). In controls, the maximum CSA was positioned in a similar distance in both sides (15.9±7.6 vs. 20.0±2.6 cm from the pubic symphysis, non-dominant and dominant sides, respectively, P = 0.19).

**Table 4:** *Rectus abdominis* cross sectional areas (values expressed in cm^2^, mean ± SD) and asymmetries.

Segments	Tennis Players					Controls						
	Dominant	Non-dominant		Total	Asymmetry(%)	Dominant	Non-dominant		Total			Asymmetry(%)
S1	10.4±2.7	12.8±3.6	*P = 0.002*	23.2±6.1	24	8.6±0.7	8.5±1.7	*P = 0.84*	17.1±2.3			-2
S2	9.9±0.9	11.8±2.6	*P = 0.03*	21.7±3.4	20	8.2±1.9	7.6±1.8	*P = 0.11*	15.8±3.6			-7
S3	8.0±2.3	10.8±2.7	*P<0.001*	18.8±4.8	38	7.6±1.5	7.0±1.2	*P = 0.46*	14.6±1.7			-5
S4	9.1±1.0	12.4±2.9	*P = 0.004*	21.5±3.7	36	7.7±1.2	7.7±1.5	*P = 0.84*	15.4±2.7			0
S5	9.0±1.2	12.8±2.5	*P = 0.001*	21.9±3.4	42	7.5±1.1	8.0±1.4	*P = 0.33*	15.5±2.3			6
S6	9.4±1.7	12.8±3.0	*P = 0.002*	22.2±4.4	37	7.4±1.1	7.9±1.2	*P = 0.27*	15.2±2.2			7
S7	8.3±1.9	12.1±3.5	*P = 0.003*	20.4±5.1	47	7.1±1.2	7.7±1.0	*P = 0.006*	14.7±2.1			8
S8	4.7±2.4	7.3±2.4	*P = 0.01*	12.0±4.4	78	4.0±2.0	5.0±1.8	*P = 0.001*	9.0	±	3.8	33

Comparisons are made into each group between dominant and non-dominant sides.

A positive relationship was observed between muscle length starting from the inter-discal L1-L2 space and the degree of asymmetry in CSA expressed as the non-dominant/dominant ratio in TP (r = 0.85, P = 0.007) and in controls (r = 0.84, P = 0.01), being more asymmetric the more distal segments.

### Differences between groups

**Figure 3:**
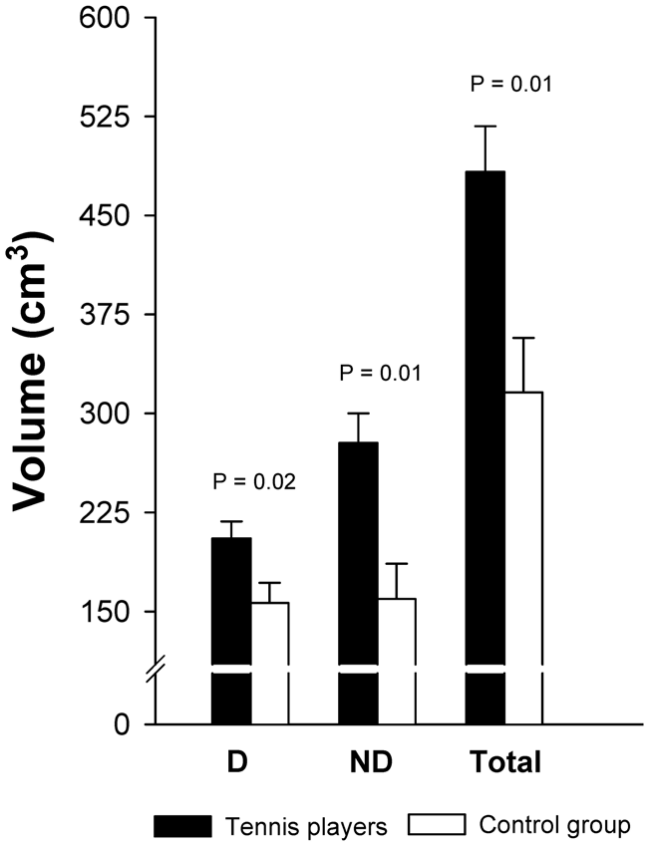
*Rectus abdominis* muscle volumes in professional tennis players and non-active subjects, after adjustment for the length of the *rectus abdominis* muscle, age and BMI.

Muscle volume of RA muscle was 52% greater in tennis players than in the control group (P = 0.003). Compared to controls, tennis players had 29% (P = 0.02) and 74% (P = 0.002) more muscle volume in the dominant and non-dominant sides, respectively. After accounting for age, the length of the RA muscle and BMI as covariates the volume of RA muscle was 58% greater in tennis players than in the control group (P = 0.01), and compared to controls, tennis players had 34% (P = 0.02) and 82% (P = 0.01) more muscle volume in the dominant and non-dominant sides, respectively (Fig. 3).

**Figure 4:**
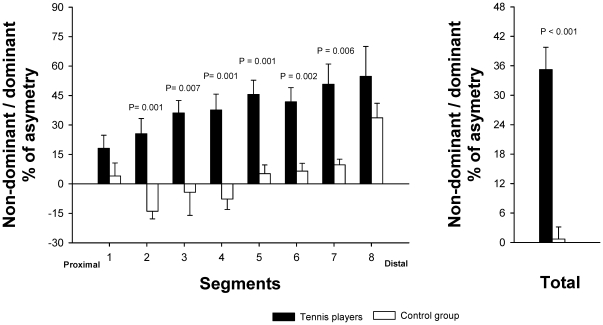
Differences between professional tennis players and non-active subjects in the percentage of asymmetry in muscle volume of *rectus abdominis*, (A) segment by segment and (B) total.

The ratio (non-dominant-dominant RA volume) x 100/dominant RA volume was greater in tennis players than in controls (35.2±12.9 vs. 0.7±6.1%, respectively, P<0.001). Between groups differences in the degree of asymmetry were statistically significant for segments 2 to 7 (Fig. 4).

In the tennis players, the maximum CSA of the dominant (P = 0.064) and non-dominant (P = 0.005) sides was greater than in controls, even after accounting for age, the length of the RA muscle and BMI as covariates (P = 0.05 and P = 0.02, respectively).

## Discussion

In this study we have determined for the first time the volume of the musculus *rectus abdominis* in professional male tennis players and comparable sedentary subjects. Tennis was associated with 58% greater *rectus abdominis* volume (both sides considered together). In addition, this study shows that in tennis players the non-dominant side of the *rectus abdominis* has a 35% greater volume that the opposed side. This contrasts with similar volumes for both sides of the *rectus abdominis* in non-active controls. However, in both groups the degree of asymmetry increased linearly from the origin (proximal) to the insertion (distal), with a similar slope. This implies that tennis appears to only exaggerate this asymmetry without altering the pattern of the side-to-side relationship observed in the controls.

Several studies have demonstrated that tennis practice increases the muscle mass and muscle volume of the dominant compared to the non-dominant arm in professional tennis players [1], [2], and that this adaptation occurs very early in life [22], [23]. In professional tennis players, inter-arm asymmetry in muscle volume is less than half the side-to-side difference in *rectus abdominis* muscle volume [2]. Assuming that this asymmetry is the result of tennis participation, it could reflect either a greater adaptation to highly asymmetrical mechanical load (even more than that observed for the arm muscles) or less likely that the *rectus abdominis* has a greater potential for hypertrophy than the muscles of the arm. Muscle size is a major determinant of the force generating capacity [24] and muscle volume a main determinant of peak power [25]. Thus, our results are compatible with a very high load on the non-dominant *rectus abdominis* which requires a higher level of hypertrophy as the muscle approaches its distal insertion. In support, several studies using electromyography highlight the importance of the *rectus abdominis* for power generation during tennis strokes, particularly when serving [4], [26]. During the serve, the *rectus abdominis* together with the *external* and the *internal* oblique muscles are submitted to a stretch-shortening cycle which is repeated several times with intention of applying maximal power to the racket [3], [27]. The RA asymmetry is likely the results of the combination of extension-flexion movements with torsion, performed predominantly in one direction. A powerful concentric contraction of RA causing trunk flexion and diagonal “shoulder to shoulder” rotation in the direction of the non-dominant side, allows the acceleration of the body before ball impact [3], [27]. To our knowledge, tennis serve is the only tennis stroke where RA activates asymmetrically [4]. No significant side-to-side differences in RA activation have been reported during the forehand stroke [26] or during side medicine-ball throw, a similar movement to forehand stroke [28].

Asymmetry of *rectus abdominis* muscle and asymmetry in trunk strength has been associated with muscle strains and lower back pain [10], [29]. In tennis players, *rectus abdominis* muscle fibril disruptions tend to occur along the deep epimysial surface below the umbilicus [17], [18], i.e. close to the region of the maximum CSA. It has been suggested that this is a potential site of weakness because the muscle is not protected by a tendinous intersection [17], [18]. Our results, are also compatible with an alternative explanation, i.e. that injuries occur in this region due to high stretch-shortening loads which may be combined with torsional strain, as reflected by the marked hypertrophy observed in this area. A recent study using MRI and sonographic images showed that competitive tennis players with and without *rectus abdominis* muscle strain injuries had a greater antero-posterior diameter in the non-dominant compared to the dominant side at the umbilical level, being greater in injured players (55% and 25% asymmetry in symptomatic and asymptomatic players, respectively) [17].

The asymmetric hypertrophy of *rectus abdominis* in both the transverse and the longitudinal axis shows that tennis elicits differential muscle activity patterns between dominant and non-dominant sides and also between upper and lower regions of the *rectus abdominis* muscle. Recent studies support the neuromuscular independence between upper and lower *rectus abdominis* [30]. Our results concur with these findings, since a different pattern of adaptation between regions of the *rectus abdominis* can only be the result of a different pattern of recruitment. The fact that *rectus abdominis* is uniquely a trunk flexor, due to the vertical orientation of the fascicles [31], makes this finding specially interesting. Differential activation have been previously reported only in muscles where fascicles change orientation, and thus function, in the different portions of the muscle, i.e. the *external oblique* and *transverse abdominis* muscles [32], [33].

To our knowledge, this is the first study that has measured the muscle volume of *rectus abdominis* in healthy humans. Therefore, we only can compare our results with a few studies analyzing CSA of *rectus abdominis* muscle using MRI or sonography in non-active subjects [19], [34], and subjects involved in different sports [14], [35]–[37]. All of these studies used images near the umbilicus to measure the CSA, which corresponds to distances between 3.5 and 13.5 cm above the pubic symphysis. In subjects slightly taller than ours (+8 cm), Hides et al. [34] reported averaged CSA for the left and right sides of 7.6 and 7.8 cm^2^, respectively, which are comparable to the areas measured in the present investigation in the non-active group (7.6 cm^2^ for both, dominant and non-dominant sides). Also, Rankin et. al [19] found average CSA in both sides of RA (8.3 and 8.2 cm^2^, right and left sides, respectively) in subjects moderately active (involved 4 days a week in recreational sports) and slightly taller than our control group (+3 cm). On the other hand, the tennis players of our study had similar total CSA (both sides added) than elite wrestlers [36], [37] and judokas [36] (21, 21 and 19 cm^2^, tennis players from the present study, wrestlers and judokas, respectively). Taking the non-dominant side only, our tennis players had a greater level of hypertrophy than elite wrestlers and judokas [36], [37]. We have estimated that had the dimensions of the non-dominant side of the tennis players been matched by the dominant side, then the total CSA of our tennis players would have been 24 cm^2^ (i.e., about 14 and 26% greater than observed in elite wrestlers and judokas, respectively). Thus, it seems that the pattern of loading elicited by tennis on the non-dominant side of the *rectus abdominis* (stretch-shortening plus torsion), could be a greater stimulus for muscle hypertrophy than that elicited by other sports.

In summary, we have shown that tennis participation at professional level is associated with 58% greater *rectus abdominis* volume (both sides considered together compared to non-athletes). Tennis players also have a marked side-to-side asymmetry due to a higher hypertrophy of the non-dominant side (35%). This contrasts with a similar RA muscle volume in both sides in non-active subjects. It remains to be determined if the side-to-side disequilibrium described in this article contributes to raise the risk of injury and back pain in tennis players.

## References

[1] Calbet JA, Moysi JS, Dorado C, Rodriguez LP (1998). Bone mineral content and density in professional tennis players.. Calcif Tissue Int.

[2] Sanchis-Moysi J, Idoate F, Olmedillas H, Guadalupe-Grau A, Alayon S (2010). The upper extremity of the professional tennis player: muscle volumes, fiber-type distribution and muscle strength.. Scand J Med Sci Sports.

[3] Elliott B (2006). Biomechanics and tennis.. Br J Sports Med.

[4] Chow JW, Park SA, Tillman MD (2009). Lower trunk kinematics and muscle activity during different types of tennis serves.. Sports Med Arthrosc Rehabil Ther Technol.

[5] Norris CM (1993). Abdominal muscle training in sport.. Br J Sports Med.

[6] David P, Mora I, Perot C (2008). Neuromuscular efficiency of the rectus abdominis differs with gender and sport practice.. J Strength Cond Res.

[7] Andersson E, Sward L, Thorstensson A (1988). Trunk muscle strength in athletes.. Med Sci Sports Exerc.

[8] Roetert EP, McCormick SWB, Ellenbecker TS (1996). Relationship between isokineticand functional trunk strength in elite junior tennis players.. Isokinetics and Exercise Science.

[9] Timm K (1995). Clinical applications of a normative data base for the Cibex TEF and TORSO spinal isokinetic dynamometers.. Isokinetic and Exercise Sciences.

[10] Hutchinson MR, Laprade RF, Burnett QM, Moss R, Terpstra J (1995). Injury surveillance at the USTA Boys' Tennis Championships: a 6-yr study.. Med Sci Sports Exerc.

[11] Barker KL, Shamley DR, Jackson D (2004). Changes in the cross-sectional area of multifidus and psoas in patients with unilateral back pain: the relationship to pain and disability.. Spine (Phila Pa 1976).

[12] Barker PJ, Guggenheimer KT, Grkovic I, Briggs CA, Jones DC (2006). Effects of tensioning the lumbar fasciae on segmental stiffness during flexion and extension: Young Investigator Award winner.. Spine (Phila Pa 1976).

[13] Hainline B (1995). Low back injury.. Clin Sports Med.

[14] Hides J, Stanton W, Freke M, Wilson S, McMahon S (2008). MRI study of the size, symmetry and function of the trunk muscles among elite cricketers with and without low back pain.. Br J Sports Med.

[15] Hides J, Wilson S, Stanton W, McMahon S, Keto H (2006). An MRI investigation into the function of the transversus abdominis muscle during “drawing-in” of the abdominal wall.. Spine (Phila Pa 1976).

[16] Hodges PW, Eriksson AE, Shirley D, Gandevia SC (2005). Intra-abdominal pressure increases stiffness of the lumbar spine.. J Biomech.

[17] Connell D, Ali K, Javid M, Bell P, Batt M (2006). Sonography and MRI of rectus abdominis muscle strain in elite tennis players.. AJR Am J Roentgenol.

[18] Maquirriain J, Ghisi JP, Kokalj AM (2007). Rectus abdominis muscle strains in tennis players.. Br J Sports Med.

[19] Rankin G, Stokes M, Newham DJ (2006). Abdominal muscle size and symmetry in normal subjects.. Muscle Nerve.

[20] Lee RC, Wang Z, Heo M, Ross R, Janssen I (2000). Total-body skeletal muscle mass: development and cross-validation of anthropometric prediction models.. Am J Clin Nutr.

[21] Bancroft LW, Peterson JJ, Kransdorf MJ, Berquist TH, O'Connor MI (2007). Compartmental anatomy relevant to biopsy planning.. Semin Musculoskelet Radiol.

[22] Sanchis-Moysi J, Dorado C, Olmedillas H, Serrano-Sanchez JA, Calbet JA (2010). Bone mass in prepubertal tennis players.. Int J Sports Med.

[23] Sanchis-Moysi J, Dorado C, Olmedillas H, Serrano-Sanchez JA, Calbet JA (2010). Bone and lean mass inter-arm asymmetries in young male tennis players depend on training frequency.. Eur J Appl Physiol.

[24] Kanehisa H, Ikegawa S, Fukunaga T (1994). Comparison of muscle cross-sectional area and strength between untrained women and men.. Eur J Appl Physiol Occup Physiol.

[25] Perez-Gomez J, Rodriguez GV, Ara I, Olmedillas H, Chavarren J (2008). Role of muscle mass on sprint performance: gender differences?. Eur J Appl Physiol.

[26] Knudson D, Blackwell J (2000). Trunk muscle activation in open stance and square stance tennis forehands.. Int J Sports Med.

[27] Lehman RC (1988). Thoracoabdominal musculoskeletal injuries in racquet sports.. Clin Sports Med.

[28] Ikeda Y, Miyatsuji K, Kawabata K, Fuchimoto T, Ito A (2009). Analysis of trunk muscle activity in the side medicine-ball throw.. J Strength Cond Res.

[29] Alyas F, Turner M, Connell D (2007). MRI findings in the lumbar spines of asymptomatic, adolescent, elite tennis players.. Br J Sports Med.

[30] Moreside JM, Vera-Garcia FJ, McGill SM (2008). Neuromuscular independence of abdominal wall muscles as demonstrated by middle-eastern style dancers.. J Electromyogr Kinesiol.

[31] Askar OM (1977). Surgical anatomy of the aponeurotic expansions of the anterior abdominal wall.. Ann R Coll Surg Engl.

[32] Mirka G, Kelaher D, Baker A, Harrison A, Davis J (1997). Selective activation of the external oblique musculature during axial torque production.. Clin Biomech (Bristol, Avon).

[33] Urquhart DM, Hodges PW, Allen TJ, Story IH (2005). Abdominal muscle recruitment during a range of voluntary exercises.. Man Ther.

[34] Hides JA, Belavy DL, Stanton W, Wilson SJ, Rittweger J (2007). Magnetic resonance imaging assessment of trunk muscles during prolonged bed rest.. Spine (Phila Pa 1976).

[35] Kubo T, Muramatsu M, Hoshikawa Y, Kanehisa H (2010). Profiles of trunk and thigh muscularity in youth and professional soccer players.. J Strength Cond Res.

[36] Iwai K, Okada T, Nakazato K, Fujimoto H, Yamamoto Y (2008). Sport-specific characteristics of trunk muscles in collegiate wrestlers and judokas.. J Strength Cond Res.

[37] Kubo J, Ohta A, Takahashi H, Kukidome T, Funato K (2007). The development of trunk muscles in male wrestlers assessed by magnetic resonance imaging.. J Strength Cond Res.

